# Melanoma lymph node metastases – moving beyond quantity in clinical trial design and contemporary practice

**DOI:** 10.3389/fonc.2022.1021057

**Published:** 2022-10-14

**Authors:** Kristen E. Rhodin, Denisse Porras Fimbres, Danielle N. Burner, Shayna Hollander, Margaret H. O’Connor, Georgia M. Beasley

**Affiliations:** ^1^ Department of Surgery, Duke University Medical Center, Durham, NC, United States; ^2^ Duke University School of Medicine, Durham, NC, United States

**Keywords:** melanoma, lymph nodes, metastasis, clinical trials, immunotherapy

## Abstract

The presence of lymph node metastases is a well-studied prognostic factor for cutaneous melanoma. Characterization of melanoma lymph node metastases and their association with survival in multiple, large observational studies has led to recognition of the following high-risk features: quantity of lymph node metastases (number of nodes), size of the nodal tumor deposit (in mm), and extracapsular extension. Despite increasing utilization of these features in the design of randomized clinical trials, in addition to their role in contemporary clinical decision-making, current staging systems lag behind, only accounting for the quantity of lymph nodes with metastases. Herein, we review the prognostic role of melanoma lymph node metastases and their high-risk features, current reporting standards, how such features have been utilized in practice-changing trials, and best practices for future clinical trial design and clinical decision-making.

## Introduction

Cutaneous melanoma remains the most aggressive and lethal form of skin cancer. Over the past few decades, the incidence of melanoma has been steadily increasing and in 2022 alone there are projected to be nearly 100,000 new cases diagnosed, with over 7% of those cases resulting in mortality (1). While most patients with cutaneous melanoma will have early-stage disease managed with surgical excision alone, outcomes are closely linked to the extent of disease and presence of high-risk markers within the primary tumor and lymph nodes. Localized melanoma that is detected early can have a 99% five-year survival rate; however, regional melanoma and metastatic disease portend a worse prognosis with 68% and 30% five-year survival, respectively (2).

Cutaneous melanoma begins with uncontrolled proliferation of melanocytes and spreads through the lymphatic system. While some patients may develop satellite or in transit lesions arising between the primary tumor and draining nodal basin, the regional lymph nodes are often the first site of metastasis. Several landmark studies have guided recommendations on nodal staging, and in contemporary practice nodal status is evaluated clinically, and often pathologically with sentinel lymph node biopsy (SLNB), at the time of diagnosis (3). Current American Joint Committee on Cancer (AJCC) staging guidelines incorporate nodal status which is defined by the number of nodes with metastatic melanoma, in addition to whether they are clinically occult or apparent (4). However, in clinical trials and practice, higher level of detail is utilized to help inform recurrence risk and guide treatment. These include: the presence of melanoma within the regional lymph nodes, the extent of involvement as measured by quantity, the size of the nodal tumor deposit, and invasion (5). In this new age of effective systemic therapies, such as immune checkpoint inhibitors (IO) or those targeted to *BRAF* mutations, determination of reliable high-risk features to aid in clinical decision-making is essential. In this review article, we describe the important prognostic information gained from lymph nodes, high-risk features of melanoma lymph node metastases, how these features are currently utilized in practice and clinical trials, as well as best practices for future study development and therapeutic decision-making.

## Prognostic features of melanoma lymph node metastases

**Figure 1:**
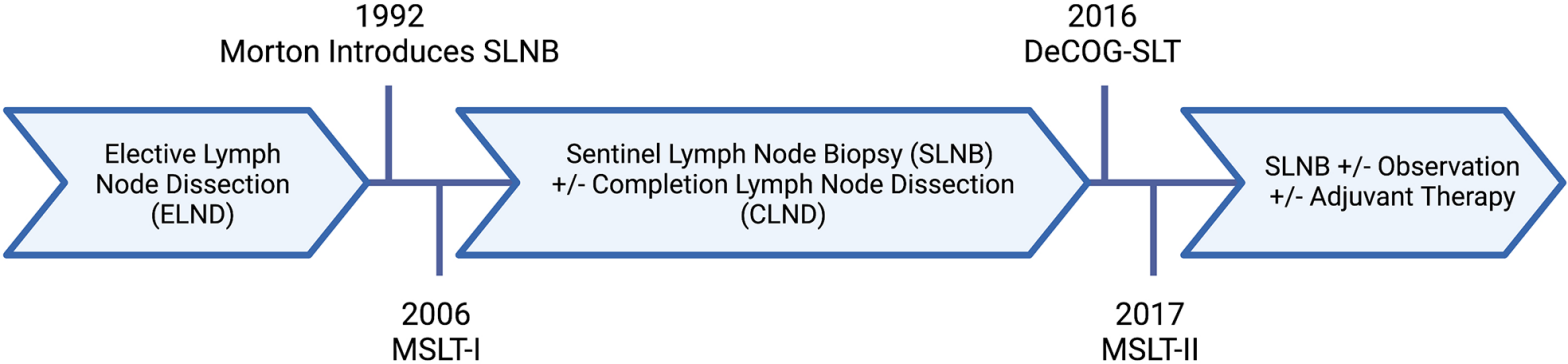
Evolution of nodal surgery for melanoma. Created with BioRender.com.

The extent of surgery for regional lymph nodes in patients with malignant melanoma, as well as our understanding of their prognostic features, has evolved over the last thirty years (**Figure 1**) (6). Traditionally, melanoma lymph node metastases were classified as “microscopic” or “macroscopic.” In the current staging system, this terminology was redefined as clinically occult or apparent, representing the method of identification of such metastases. Clinically occult lymph node metastases are identified through SLNB; whereas, clinically apparent lymph node metastases are detected by clinical or radiographic examination. This distinction has implications on survival for stage III melanoma, with an abundance of evidence demonstrating better outcomes for patients with clinically occult regional nodal disease (4).

For melanoma patients without clinically apparent nodal disease, sentinel lymph node (SLN) status has historically been the most important predictor of survival. In 2001 the AJCC incorporated the N1-2a criteria into the staging system for cutaneous melanoma. However, it has been consistently observed that sub-groups of SLN-positive patients can have very different survival rates, ranging from as low as 30% to over 90% (7–9). Indeed, positive SLNs are heterogeneous and should not be treated as a binary prognostic feature. As such, further investigation into which characteristics of a positive SLN most influence a patient’s risk of recurrence and poor outcomes has been of great interest.

### Sentinel lymph node disease burden (quantity and size)

While not a feature of the SLN itself, positive non-SLN status – that is, the presence of micro- or macro-metastasis within nodes outside of the primary draining lymph node – is typically associated with a poorer prognosis. With this in mind, Gershenwald et al. developed a model stratifying patients according to their risk for non-SLN involvement based on a retrospective analysis of 2,203 patients with clinically node-negative melanoma who underwent SLNB and were found to have a positive SLN. The authors concluded that SLN tumor burden (i.e. size of the metastatic tumor, the number of involved lymph nodes, and the presence or absence of extracapsular extension), the number of SLNs harvested, and the primary tumor thickness were the strongest predictors of synchronous non-SLN involvement in melanoma patients with positive SLNs (10). Their stratification of largest tumor focus in the SLN suggested increasing risk beyond 2 mm; however, this dimension was not analyzed as a continuous variable (10).

Another retrospective review of 2,313 patients with stage III melanoma by Balch et al., confirmed marked heterogeneity in five-year survival, particularly for patients with microscopic (clinically occult disease) versus macroscopic (clinically or radiologically identified, pathologically confirmed) nodal disease. In multivariable analysis, they identified the quantity of lymph node metastases as the most significant independent predictor of survival (9). Of note, the authors did not report the size of nodal tumor deposits nor stratify further than micro- and macroscopic nodal burden (9).

Further stratification of the size of nodal tumor deposits has been described. A retrospective study by van Akkooi et al. examined 388 patients with positive SLN and utilized the Rotterdam classification for tumor burden and maximum diameter (<0.1 mm, 0.1-1mm, >1mm) (11). They report similar outcomes to SLN-negative patients for those with nodal tumor deposits less than 0.1 mm and the least favorable prognosis for patients with greater than 1 mm nodal tumor deposits (11). Later, van der Ploeg et al. conducted an international, multicenter retrospective study of 1,539 sentinel node-positive melanoma patients that evaluated the indices of SLN tumor burden such as intranodal location, tumor penetrative depth (TPD) and maximum size of nodal tumor deposits (8). They also utilized the Rotterdam classification and found nodal tumor deposit greater than 1 mm to be predictive of non-SLN positivity and associated with poor melanoma specific survival (MSS) (HR 3.55, 95% CI 2.17-5.80; p<0.001) (8). Additional factors of interest included non-subcapsular location (i.e. extracapsular extension [ECE]) and increasing TPD (8).

Importantly, investigators have begun to evaluate these prognostic features of positive SLNs in the “modern era” of melanoma management, where patients are no longer receiving completion lymph node dissection (CLND) and there are effective adjuvant therapies available. Mitra et al. report a retrospective analysis of 215 SLN-positive melanoma patients who did not undergo CLND. Of this cohort, approximately half of these patients received adjuvant therapy and of those, almost all patients received immunotherapy (12). Even in the setting of extensive systemic therapy, the SLN basin was the most common site of recurrence in 67% of patients that relapsed (12). On multivariable analysis, both the quantity of lymph nodes with melanoma metastases as well as nodal deposits greater than 1 mm were independently associated with recurrence (12).

Of note, patients with stage IIIA melanoma who have positive SLN are generally only considered for adjuvant therapies if the nodal tumor deposit is greater than 1 mm. In their recent publication, Moncrief et al. compared 408 patients with stage IIIA melanoma to 3,199 patients with stage IB, whose final staging only differs in the involvement of a positive SLN (13). Utilizing maximally selected rank statistics, they identified a nodal tumor deposit dimension of 0.3 mm as a cutoff point in recurrence risk and survival (overall, disease-specific, and distant-metastasis free) and validated this in a multivariable model (HR 1.26, 95% CI 1.11-1.44; p<0.0001) (13). Therefore, proposing that patients with stage IIIA melanoma and <0.3 mm nodal tumor deposit may be treated similarly to their negative SLN counterparts. More importantly, their findings expand the literature on prognostic features of the SLN and may have implications on provision of adjuvant therapy.

### Extracapsular extension

An additional prognostic feature of melanoma lymph nodes metastases is extracapsular extension (ECE). As described by the Bhattacharya et al., “lymph node extracapsular extension, also known as extranodal extension or extracapsular spread (ECS) from lymph nodes, is defined as metastatic cancer extending through the nodal capsule into the peri-nodal adipose tissue and is a hallmark of aggressive phenotype for multiple cancers.” (14) As reported by articles previously mentioned, non-subcapsular location is independently associated with poorer melanoma-specific survival (8). For patients with ECE, the possibility of microscopic tumor cells in the surrounding stroma even in the setting of lymphadenectomy remains high and recurrence in the SLN basin may be a natural outgrowth of these residual cells.

An early retrospective study of 338 patients in 2000 by Pidhorecky et al. demonstrated increased recurrence risk with increasing number of involved lymph nodes and extracapsular extension (ECE) following elective and therapeutic lymph node dissections (15). Khosrotehrani et al. expanded upon this study with creation of a nomogram for predicting recurrence in patients with stage IIIB/C melanoma after lymph node dissection. Their nomogram highlights the prognostic significance for both the number of positive nodes and ECE, which was subsequently validated in a separate cohort (16).

While ECE has traditionally been a high-risk feature for macro-metastatic, or clinically evident disease in the lymph nodes, there has been increasing interest in the prognostic utility of ECE for positive SLNs. In their single institution, retrospective study, Lobo et al. describe an association between ECE and increased metastasis in addition to worse overall survival (17). Another retrospective analysis by Lo et al. compared the outcomes for patients with micro- and macro-metastatic lymph node disease with or without ECE. They report similar disease-specific survival (DSS) for patients with ECE and positive SLNs compared to macro-metastatic disease in the nodes without ECE (18). On multivariable analysis ECE was an independent predictor of DSS, OS, and progression-free survival in patients with positive SLNs, further supporting its use as a prognostic factor in the micro-metastatic setting (18).

### Lymph node ratio

The Lymph Node Ratio (LNR)—the number of metastatic lymph nodes (LNs) over the total number of excised LNs after lymphadenectomy—is a prognostic factor for many solid tumors but remains controversial in the melanoma community with varying supporting literature. For instance, Grotz et al. sought to explore the prognostic utility of the LNR in a retrospective cohort analysis of 411 patients with stage III melanoma. While the authors were able to show that LNR, non-SLN involvement, ECE, macro-metastasis, and N stage were important prognostic factors in stage III melanoma following complete LN dissection, LNR alone failed to significantly improve stratification over the current AJCC staging system in univariate analysis (19).

**Figure 2:**
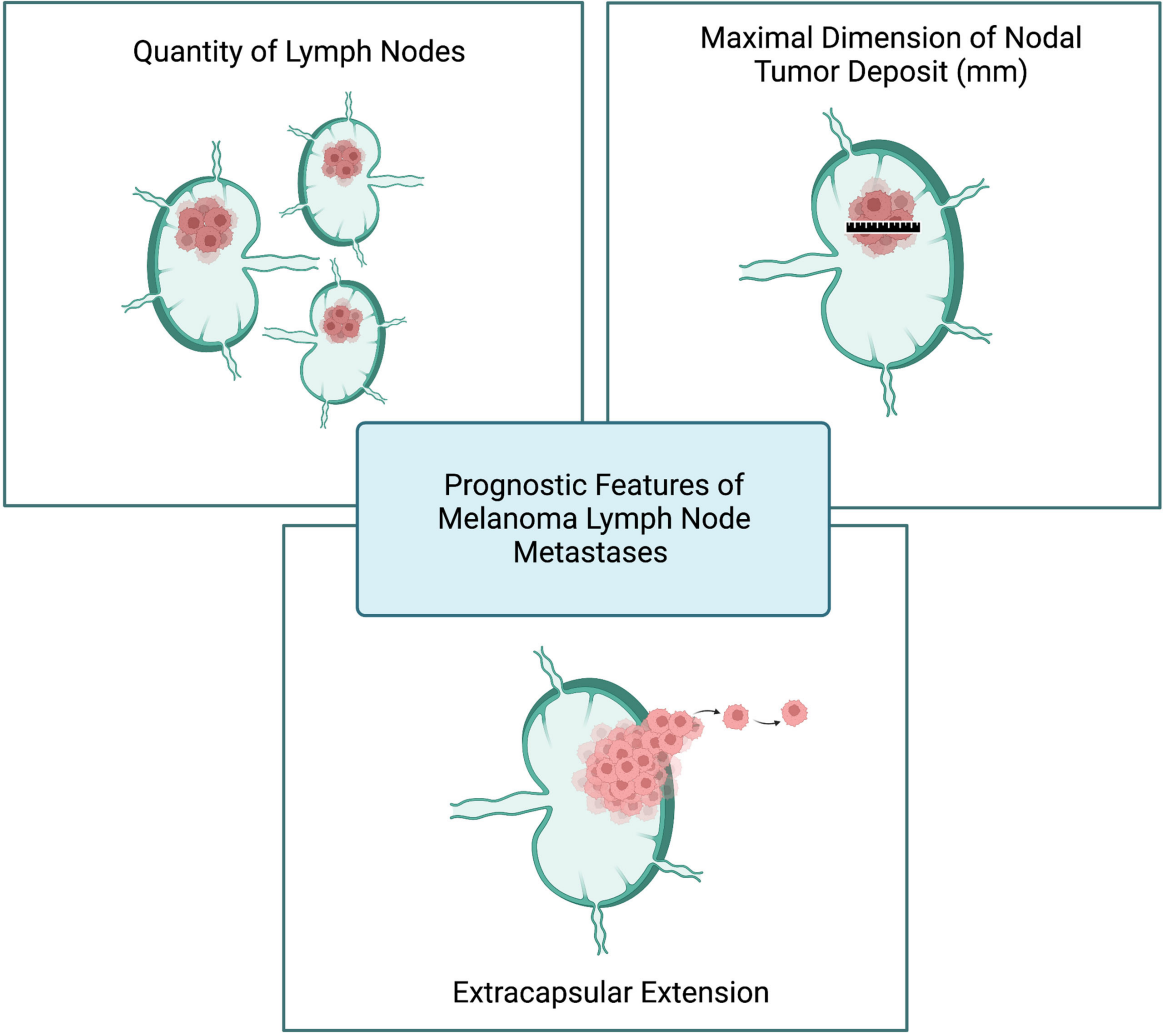
Prognostic features of melanoma lymph node metastases. Created with BioRender.com.

To further study LNR as a prognostic feature, Sandro et al. conducted a large retrospective analysis of 2,526 melanoma patients with LN metastasis. In contrast to Grotz et al, the LNR was found to be a predictive of melanoma-specific survival on univariate analysis (20). The authors went on to assess the utility of merging together the AJCC N substages and the LNR and their data ultimately suggest a prognostic role for LNR in melanoma patients with one (AJCC N1a) and two to three (AJCC N2a) positive LNs after SLNB (20). Given the conflicting evidence on its utility, LNR has yet to be incorporated into pathology reports or clinical trial study design. Rather, the quantity of lymph node metastases, nodal tumor deposit dimension, and presence of ECE are considered the core prognostic features of melanoma lymph node metastases (**Figure 2**).

## Contemporary reporting of lymph node features

Despite mounting literature on prognostic features of melanoma lymph node metastases, contemporary staging only accounts for the number of lymph nodes with metastatic melanoma and whether they were clinically detected. The AJCC criteria for melanoma staging, which was most recently revised in 2018, uses the standard tumor, nodes, and metastatic prognostic factors with the addition of anatomic factors to improve assessment (4, 21). In the AJCC 8^th^ edition, N still signifies the number of tumor-involved regional lymph nodes to measure nodal metastatic burden similar to prior editions. A notable change to the nodal criterion is the inclusion of microsatellite, satellite, and in-transit non-nodal regional metastasis, which have been associated with adverse prognosis (4). Although such staging changes were made to reflect greater prognostic accuracy, they lack granularity in lymph node prognostic features, particularly for clinically occult lymph node metastases.

Although the current staging system lags in recognizing such prognostic features, standards for reporting these high-risk features have been established. For SLN, the National Comprehensive Cancer Network (NCCN) recommends recording the number of positive and negative sentinel and non-sentinel lymph nodes examined (5). If metastases are present, the greatest dimension of tumor size (in mm and measured to the nearest 0.1 mm), location within the node, and presence of ECE should be recorded (5). Standard pathologic reporting of such features is crucial to multidisciplinary assessment of an individual patient’s recurrence risk and for creating a treatment or surveillance plan in contemporary melanoma management.

In addition to national guidelines, high-risk features of melanoma lymph node metastases have been utilized in the design of large, randomized clinical trials, including those examining de-escalation of nodal surgery. In the German Dermatologic Cooperative Oncology Group trial (DeCOG-SLT), a tumor thickness of at least 1 mm and micro-metastasis in the SLN were inclusion criteria. Notable exclusion criteria were macro-metastasis (>2mm nodal tumor deposit) or ECE (6, 22). Similarly, the second multi-center selective lymphadenectomy trial (MSLT-II) excluded patients with ECE of the nodal tumor deposit recognizing its worse prognosis; however, they did not exclude based on the dimension of the nodal tumor deposit (23). Interestingly, neither study specified inclusion nor exclusion criteria based on the quantity of positive lymph nodes, even though this is the only prognostic feature used in staging. Further, DeCOG-SLT and MSLT-II did not find a relationship between the number of positive SLN and survival; however high SLN tumor burden (>1 mm) was a significant prognostic factor (6, 22, 23). Over half of patients in both trials had metastases ≤1 mm and therefore were unlikely to be at risk for additional, non-sentinel nodal metastasis (22, 23).

During accrual for the surgical de-escalation trials, systemic therapy for melanoma evolved and trials examining both immune checkpoint inhibitors (ICI) and targeted therapies in the adjuvant setting were initiated. Many of these trials incorporated high-risk features of lymph nodes metastases into their design. The initial adjuvant ICI trial, EORTC 18071 demonstrated increased 3-year RFS, OS, and DMFS for patients treated with ipilimumab (anti-CTLA-4) after resection of stage III melanoma, although 41.6% of patients experienced adverse events, leading to discontinuation in half of patients (24). Given the risk associated with nodal tumor deposits greater than 1 mm, EORTC 18071 inclusion criteria utilized this cutoff for patients with stage IIIA (N1a) disease (24). Alternatively, Checkmate 238 which compared adjuvant nivolumab (anti-PD1) to ipilimumab utilized staging in their inclusion criteria (stage IIIB and greater), completely excluding stage IIIA (25). Checkmate 238 reported that nivolumab increased RFS compared to ipilimumab and resulted in lower toxicity for stage III and IV melanoma (25). Concurrently, two separate studies – Keynote 054 (EORTC 1325), examining adjuvant pembrolizumab (anti-PD1); and COMBI-AD, examining adjuvant dabrafenib/trametinib (BRAF/MEK inhibitors) – returned to the Rotterdam criteria, necessitating at least 1 mm nodal tumor deposit for patients with stage IIIA disease (26, 27). Both studies demonstrated prolonged RFS for patients receiving the respective adjuvant agent compared to placebo (26, 27).

Given the inclusion criteria of these trials, NCCN guidelines recommend consideration of adjuvant ICI or targeted therapy for patients with cutaneous melanoma and pathologic involvement of lymph nodes (of more than 1 mm in dimension) who have undergone complete resection (5). Decision-making regarding adjuvant therapy is undoubtedly complex; however, increasing prevalence of these high-risk features in clinical trial design and national guidelines validates their utility in risk-stratification. As the premise of staging systems lies in such risk-stratification, perhaps it is time to factor in such prognostic features to the melanoma N staging system. Inclusion of such factors may facilitate more specific clinical trial design, improved generalizability of such trial results, and standardize their applications.

## Best practices and conclusions

In conclusion, SLN positivity remains an important prognostic factor in melanoma; however, its prognostic value is much more granular than currently accounted for in the contemporary staging system. Beyond the quantity of lymph nodes involved, this review has highlighted the maximal nodal tumor deposit dimension (in mm) and presence of ECE as additional high-risk features. These additional markers have been utilized in patient selection for large randomized clinical trials, particularly the 1 mm nodal deposit cutoff of the Rotterdam criteria and are perhaps more important than stage in ongoing trials. As with prior adjuvant trials, RELATIVITY-098 (NCT05002569) a phase 3 trial comparing combination relatlimab (anti-LAG-3) and nivolumab to nivolumab alone in the adjuvant setting utilizes stage IIIA with at least 1 mm nodal tumor deposit as the threshold for inclusion. Alternatively, the MelPORT trial (NCT04594187), a phase 2 study examining post-operative nodal radiation for patients with SLN positive melanoma, does not utilize AJCC staging in their inclusion criteria of high-risk patients. Rather, their definition of high-risk includes ECE, 0.5 mm or greater nodal tumor deposit, two or greater lymph nodes with metastatic melanoma, and lymphovascular invasion of the primary tumor.

While these high-risk features are not included in the latest staging system, they are often discussed in multidisciplinary tumor boards to guide clinical management. Given the expanding literature on such high-risk features and their utilization in practice-changing trials, best practices include incorporating these prognostic features into synoptic pathology reports. Further, multidisciplinary evaluation of patients who possess such features is essential. Patients with positive SLNs are often heterogeneous and the definition of high-risk remains dynamic. Continual re-evaluation of prognostic features is critical to better defining these high-risk populations and those who will most benefit from close observation, further surgical resection, or systemic therapies. As discussed, these features will likely continue to be included in selection criteria in randomized trials. When designing clinical trials in stage III melanoma patients, specifying and collecting detailed characteristics or lymph node disease could limit heterogeneity that results from using AJCC stage alone. Consideration of these features in the next AJCC staging edition for melanoma will better align with trial data and ultimately provide better guidance for contemporary practice patterns, recurrence risk, and management recommendations.

## Author contributions

KR and GB outlined the topic. KR, DF, DB, SH, MO, and GB drafted the manuscript. All authors contributed to the article and approved the submitted version.

## Funding

KR is supported by NIH 1R38AI140297. GB is supported by NIH K08 CA237726-01A1. GB has received clinical trial funding paid to Duke University from Delcath, Istari, Replimune, Oncosec, Checkmate. GB has done one-time advisory board for Cardinal Health, Regeneron.

## Acknowledgments

**Figures 1** and **2** were created by K. Rhodin with BioRender.com.

## Conflict of interest

The authors declare that the research was conducted in the absence of any commercial or financial relationships that could be construed as a potential conflict of interest.

## Publisher’s note

All claims expressed in this article are solely those of the authors and do not necessarily represent those of their affiliated organizations, or those of the publisher, the editors and the reviewers. Any product that may be evaluated in this article, or claim that may be made by its manufacturer, is not guaranteed or endorsed by the publisher.
